# Clinical Outcomes and Molecular Epidemiology of Human Metapneumovirus in Romanian Hospitalized Patients

**DOI:** 10.3390/microorganisms14020403

**Published:** 2026-02-08

**Authors:** Ovidiu Vlaicu, Oana Săndulescu, Anca Streinu-Cercel, Anca Cristina Drăgănescu, Victor Daniel Miron

**Affiliations:** 1 National Institute for Infectious Diseases “Prof. Dr. Matei Balș”, 021105 Bucharest, Romania; 2 Faculty of Medicine, Carol Davila University of Medicine and Pharmacy, 050474 Bucharest, Romania; 3 Academy of Romanian Scientists, 050094 Bucharest, Romania

**Keywords:** human metapneumovirus, clinical outcomes, molecular genetics, hospitalized, Romania

## Abstract

Human metapneumovirus (hMPV) is an important cause of acute respiratory tract infections. This study aimed to describe the clinical characteristics, outcomes, and molecular features of hMPV infection among hospitalized patients in Romania. We performed an analysis of prospectively collected surveillance data from patients hospitalized with influenza-like illness or severe acute respiratory infection and tested by RT-PCR for the presence of respiratory viruses between November 2023 and May 2025. Only cases of hMPV monoinfection were analyzed. Clinical, laboratory, and outcome data were analyzed, and a subset of samples with high viral load underwent genetic sequencing of the hMPV fusion (F) gene. A total of 71 patients met the criteria. Children accounted for 62.0% of cases. The clinical features were nonspecific, dominated by cough (87.3%), fever (80.3%), and nasal congestion (47.9%). Adults were significantly more likely to develop dyspnea and respiratory failure requiring oxygen supplementation (51.9% vs. 6.8%, *p* < 0.001). The median length of hospital stay was 5 days (interquartile range: 2, 7 days), and dyspnea at admission was the strongest factor associated with prolongation of hospitalization. The rate of intensive care unit admission was 4.2%, and overall outcomes were favorable, with no deaths recorded. Molecular analysis revealed the circulation of different hMPV subclades across consecutive seasons, with A2b1 predominating in 2023–2024 and A2b2 in 2024–2025. hMPV infection in hospitalized patients presents with nonspecific clinical features and shows distinct age-related patterns of severity and complications. Early identification of respiratory involvement, particularly dyspnea at presentation, may support risk stratification and optimized clinical management. Preliminary molecular data indicate dynamic circulation of hMPV subclades, underscoring the value of integrated clinical and molecular surveillance. These findings support the inclusion of hMPV in the differential diagnosis of severe acute respiratory infections and highlight the importance of continued monitoring in the post-pandemic period.

## 1. Introduction

Human metapneumovirus (hMPV) remains a globally relevant cause of acute respiratory tract infections. Infants and young children, especially those under 2 years of age, are at high risk for severe lower respiratory tract disease, including bronchiolitis and pneumonia. hMPV is a leading cause of pediatric hospitalizations for acute respiratory tract infections, with increased severity in those with underlying comorbidities [1,2,3]. Furthermore, older adults (over 60 years old) are at increased risk of severe disease, hospitalization, and complications. Frailty and the presence of chronic conditions elevate this risk [4,5,6]. Also, immunocompromised individuals, including those with malignancies, organ transplantation, or immunosuppressive therapy, are at high risk for severe and complicated hMPV infection [2,3,4]. In addition, patients with chronic conditions, such as chronic obstructive pulmonary disease, congestive heart failure, coronary artery disease, diabetes mellitus, end-stage renal disease, neuromuscular disorders, and malnutrition, carry an increased risk for hospitalization and severe outcomes during hMPV infection [4,5,6]. While the clinical outline of hMPV infection has been previously described, factors such as variability in study designs, surveillance systems, diagnostic criteria, or hospitalization thresholds across different studies may affect comparability and may hinder a complete picture of the pathogenicity and severity of disease.

hMPV circulation has a peak activity typically in late winter and spring in temperate regions, and variable timing in tropical areas [7]. The impact of the coronavirus disease 2019 (COVID-19) pandemic on hMPV epidemiology was substantial. During periods of strict mitigation (lockdowns, masking, distancing), hMPV incidence dropped dramatically, with some regions reporting detection rates less than 5% of pre-pandemic levels. Following relaxation of these measures, hMPV resurged, often with atypical timing and increased incidence, including off-season outbreaks and higher rates in both pediatric and adult populations [4,7,8]. The interval between respiratory syncytial virus (RSV) and hMPV peaks also widened, and cocirculation periods shortened, impacting clinical and public health preparedness [8]. Importantly, it is unclear whether these altered patterns will persist or normalize over time, which highlights the importance of performing surveillance studies ideally through a unified methodology to allow comparability within and between countries.

The importance of diagnosis and epidemiological monitoring is underscored by the clinical overlap of hMPV with other respiratory viruses, including RSV, influenza, and severe acute respiratory syndrome coronavirus 2 (SARS-CoV-2). Accurate diagnosis enables appropriate patient management, infection control, and surveillance. Epidemiological monitoring is critical for detecting outbreaks, understanding burden, guiding resource allocation, and informing vaccine development and public health strategies. Enhanced post-pandemic surveillance is essential to characterize evolving seasonality and to optimize prevention and preparedness [4,7,8,9].

The burden of hMPV in Romania is likely underrecognized due to limited routine testing and surveillance gaps. Certainly, hMPV contributes to significant morbidity, healthcare utilization, and economic burden, as seen in other European settings [10,11]. In this context, the primary objective of our study was to describe the clinical characteristics and outcomes of patients hospitalized with hMPV infection in Romania. Additionally, for a subset of hMPV-positive samples, we aimed to perform molecular genetic characterization of the circulating strains.

## 2. Methods

We conducted an analysis of all hospitalized cases of hMPV infection identified between November 2023 and May 2025 at the National Institute for Infectious Diseases “Prof. Dr. Matei Balș” (NIID), Bucharest, Romania. NIID is the main reference center for the care, research, and coordination of infectious diseases in Romania. In addition, NIID is a participating center in the international Global Influenza Hospital Surveillance Network (GIHSN). Consequently, respiratory infection surveillance is performed prospectively in accordance with a standardized international protocol, as previously described [12,13,14,15].

### 2.1. Case Selection and Testing

In accordance with the GIHSN protocol, patients presenting at NIID with influenza-like illness (ILI) or severe acute respiratory infection (SARI) with symptom onset within the previous 7 days [12,13,14,15,16] who agreed to participate in the study, underwent nasopharyngeal swab collection using viral transport medium (Copan Italia S.p.A., Brescia, Italy). All specimens were subsequently tested using the QIAstat-Dx Respiratory Panel Plus multiplex RT-PCR assay (Qiagen, Hilden, Germany), which detects 21 viral and bacterial respiratory pathogens. (SARS-CoV-2, influenza A, influenza A H1N1 pdm09, influenza A H1, influenza A H3, influenza B, human coronaviruses 229E, HKU1, NL63 and OC43, parainfluenza viruses 1, 2, 3 and 4, adenovirus, respiratory syncytial virus, human metapneumovirus, human rhinovirus/enterovirus, *Mycoplasma pneumoniae*, *Bordetella pertussis*, and *Chlamydophila pneumoniae*).

In this study, we included only hospitalized patients admitted from 1 November 2023 onward, who met the criteria for ILI or SARI and tested positive for hMPV. Patients with respiratory co-infections, those hospitalized for less than 24 h, and those with incomplete data were excluded from the analysis.

### 2.2. Data Collection

After providing written informed consent, a standardized questionnaire was completed for each patient, collecting information on clinical signs and symptoms according to the ILI and SARI definitions, epidemiological exposure, and underlying chronic conditions. Additional data regarding laboratory test results, clinical management, and patient outcomes were extracted from medical records.

Based on age at the time of testing, patients were categorized into two groups: children (<18 years) and adults (≥18 years).

### 2.3. Genetic Analysis

Samples from eligible patients were processed for genetic sequencing of hMPV. The viral RNA was extracted from 500 μL viral transport media using QiAmp DSP Virus Kit (Qiagen, Hilden, Germany) [17], and confirmation of hMPV was performed by real-time RT-PCR using the Allplex™ Respiratory Panel 2 kit (Seegene^®^, Seoul, Republic of Korea) according to the manufacturer recommendations [18].

For hMPV F gene sequencing, samples with a high viral load (cycle threshold value (Ct) < 25) were selected to ensure the efficiency of the technique. A fragment of the hMPV F gene from hMPV-positive samples was amplified using the Qiagen One-Step RT-PCR kit (Qiagen, Hilden, Germany) using primers/protocol previously described [19]. Amplified products corresponding to nucleotide positions 41-8083 in the genome of the hMPV strain A1 (NCBI GenBank Accession KU821121) were visualized via electrophoresis on a 1% SYBR Safe DNA-stained agarose gel. Amplicons were extracted and purified with a PureLink Quick Gel Extraction kit (Invitrogen, Thermo Fisher Scientific, Waltham, MA, USA). The generated amplicons were further processed with an Illumina DNA Prep Kit (Illumina, San Diego, CA, USA) according to the manufacturer’s recommendations. Sequencing was performed on the MiSeq platform (Illumina) by using the paired-end shotgun strategy. The reads were assembled by a double approach protocol (de novo and reference mapping) as previously described [20]. A reference sequence for each viral genotype was used for mapping purposes.

hMPV F gene nucleotide sequences of reference strains with known genotype identities were retrieved from GenBank (https://www.ncbi.nlm.nih.gov/). Romanian and reference sequences were aligned using the BioEdit software (version 7.2.5) and then the resulting alignments were used as input for the generation of phylogenetic tree using the MEGA7 Tree Builder method with the following algorithm: HKY genetic distance model, Neighbor-Joining tree build method, 1000 replicates bootstrap. The phylogenetic tree was generated using a portion of hMPV F gene (591 nucleotide, 4058-4645nt).

### 2.4. Statistical Analysis

Data were analyzed using SPSS software, version 25 (IBM Corp., Armonk, NY, USA). Continuous variables were summarized as median and interquartile range (IQR, 25th–75th percentile) due to non-normal distribution, while categorical variables were expressed as frequencies (n, N) and percentages (%). Group comparisons were performed using the Mann–Whitney U test for continuous variables and the chi-square test or Fisher’s exact test, as appropriate, with odds ratios (ORs) and 95% confidence intervals (95% CIs). To identify independent predictors of length of hospital stay, a multivariable linear regression model was constructed. Variables associated with hospitalization duration in univariable analyses or considered clinically relevant were included in the multivariable model. Regression coefficients were reported as unstandardized beta coefficients (B) with corresponding standard errors and 95% confidence intervals. Model assumptions were assessed by evaluating collinearity using variance inflation factors, with values < 5 considered acceptable. Model fit was evaluated using the adjusted coefficient of determination (adjusted R^2^). A two-sided *p*-value < 0.05 was considered statistically significant.

## 3. Results

### 3.1. Clinical Characteristics

Out of a total of 4697 patients tested by RT-PCR during the study period, 1361 were positive for at least one respiratory virus. Among these, 93 patients were positive for hMPV, of whom 71 met the eligibility criteria and were included in the analysis. The highest number of hMPV cases was identified in March, both in 2024 (n = 15 cases) and in 2025 (n = 14 cases). Overall, children (62.0%, n = 44), and females (58.0%, n = 40) were predominant. The median age of the study population was 7.2 years (IQR: 2.6, 53.3 years). Among children, the median age was 3.7 years (IQR: 1.5, 7.0 years), whereas among adults it was 61.7 years (IQR: 49.8, 71.2 years). Underlying chronic conditions were significantly more frequent among adults compared with children (63.0%, n = 17/27 vs. 2.3%, n = 1/44; *p* < 0.001).

The clinical features of hMPV infection were dominated by cough (87.3%, n = 62), fever (80.3%, n = 57), and nasal congestion (47.9%, n = 34). Children were 6 times more likely (OR = 6.12) to have nasal congestion (*p* < 0.001), while adults were almost 8 times more likely to have dyspnea (OR = 0.12, *p* = 0.001), Table 1. Myalgia and headache were reported only among adults.

Blood cell and inflammatory syndrome findings are shown in Table 2. Children presented with significantly higher median white blood cell (WBC) counts compared with adults (9010 vs. 6970 cells/µL; *p* = 0.013), with a moderate effect size (*r* = 0.316). However, the proportion of patients with increased or decreased WBC values did not differ significantly between groups. Median monocyte counts were markedly higher in children than in adults (920 vs. 480 cells/µL; *p* < 0.001), corresponding to a large effect size (*r* = 0.492). Consistently, increased monocytes were significantly more frequent in children (54.5% vs. 22.2%; *p* = 0.007). Children also exhibited significantly higher lymphocyte counts compared with adults (2720 vs. 1070 cells/µL; *p* < 0.001), with a large effect size (*r* = 0.577). Despite this difference in absolute counts, the prevalence of lymphopenia was similar between groups. Adults showed significantly higher inflammatory burden, reflected by higher median CRP levels (23.7 vs. 6.9 mg/L; *p* < 0.001; *r* = 0.387). Correspondingly, elevated CRP values were more frequently observed in adults (96.3% vs. 70.5%; *p* = 0.006).

Adults with hMPV infection were significantly more likely to develop respiratory failure requiring oxygen supplementation compared with children (51.9% vs. 6.8%; OR = 0.07; *p* < 0.001; Table 3), whereas moderate to severe dehydration was five times more frequent among children (OR = 5.00; *p* < 0.001; Table 3). On imaging evaluation, interstitial pneumonia was identified in 19 cases, with a similar distribution between children and adults, while pulmonary consolidation was observed in only three cases, all occurring in adults. Only three adult patients required admission to the intensive care unit (ICU). No deaths associated with hMPV infection were recorded.

**Figure 1:**
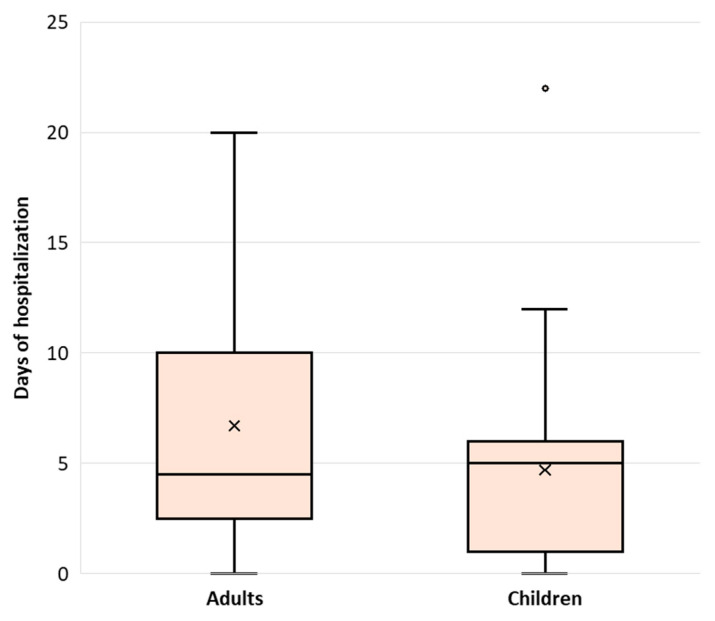
Length of hospital stay according to age groups.

**Figure 2:**
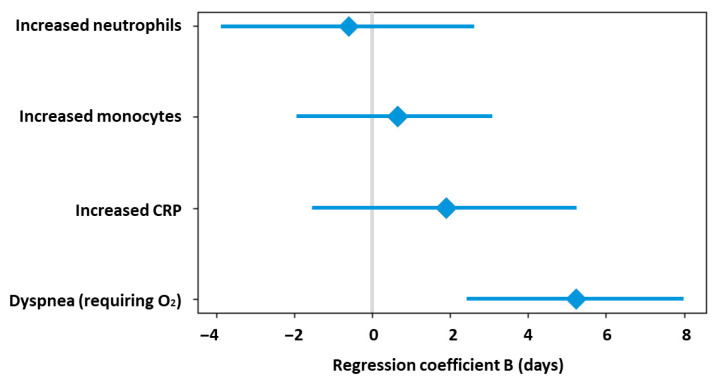
Independent predictors of length of hospital stay in patients with metapneumovirus infection.

The median length of hospital stay was 5 days (IQR: 2, 7 days), with no significant differences between adults (4.5 days, IQR: 2.5, 10 days) and children (5 days, IQR: 1, 6 days), Figure 1. In the univariate analysis of predictors of length of hospital stay, the presence of dyspnea on admission requiring oxygen (+5 days, *p* < 0.001, *r* = 0.514), increased neutrophil count (+5 days, *p* < 0.001, *r* = 0.494), increased monocyte count (+2 days, *p* = 0.011, *r* = 0.326), and increased CRP values (+2 days, *p* = 0.010, *r* = 0.371) were significantly associated with prolonged hospital stay. A multivariable linear regression model including these factors was statistically significant (adjusted R^2^ = 0.263, *p* = 0.004), but dyspnea at admission was the only independently associated factor of prolonged hospitalization, being associated with an increase of approximately 5 days in length of stay (B = 5.21, *p* = 0.001, Figure 2).

### 3.2. Molecular Characteristics

**Figure 3:**
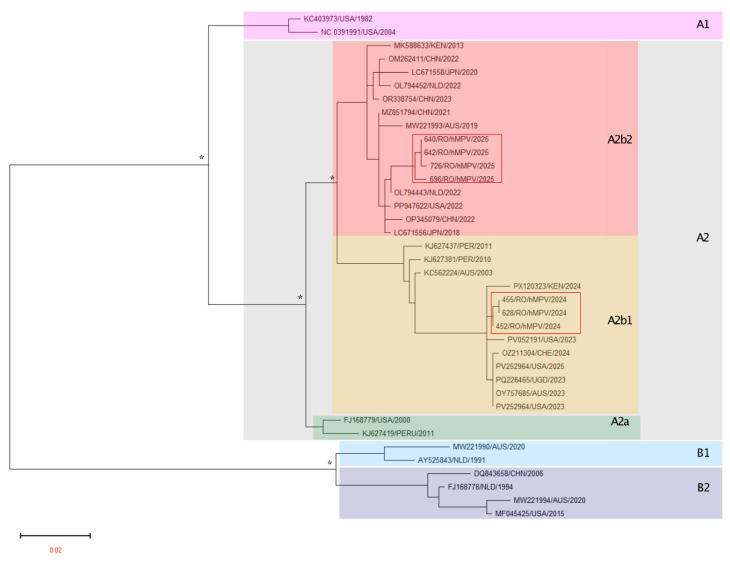
Phylogenetic analysis for hMPV Romanian samples. The red boxes highlight the strains from Romania.

Out of the 71 hMPV-positive samples, 17 had a Ct value < 25 and were selected for sequencing. Overall, seven samples were successfully sequenced. The phylogenetic analysis for Romanian showed circulation of the two major genotypes (A2b1 and A2b2, Figure 3). In total, three hMPV Romanian strains belonging to the A2b1 genotype and four to the A2b2 genotype were identified in the 2023/2024 and 2024/2025 respiratory infection season, respectively.

## 4. Discussion

In this analysis, we characterized hMPV infection among hospitalized patients treated at the largest infectious diseases hospital in Romania, addressing clinical, laboratory, and outcome characteristics, with an exploratory molecular component. In addition, our study provides initial molecular data on circulating hMPV strains detected in Romania and, to our knowledge, represents the first reported molecular characterization of clinical hMPV strains from this setting.

To accurately capture the clinical features of hMPV infection, we included only cases of monoinfection, thereby minimizing potential confounding effects related to viral or bacterial co-infections. Although the overall sample size was relatively limited, the analyzed data were derived from a rigorously standardized international surveillance and testing system applied to hospitalized patients with ILI and/or SARI. NIID has been a member of the GIHSN since 2017, and eligible patients are tested continuously throughout the year, rather than exclusively during the cold season. This approach allows for a robust description of hospitalized hMPV cases in our region.

In our study, hMPV infection was identified in both children and adults hospitalized with ILI/SARI, with the highest number of cases observed in March in both 2024 and 2025. The clinical presentation was nonspecific, being dominated by fever as a systemic symptom and by cough and nasal congestion as the main respiratory manifestations. Consequently, these clinical features largely overlap with those of other viral respiratory infections and are not specific to hMPV infection.

Building on the consensus that no clinical symptoms or signs are specific for hMPV infection, observational studies and cohort analyses further reinforce the overlap in clinical presentation between hMPV and other respiratory viruses. Across both pediatric and adult populations, hMPV most commonly manifests as fever, cough, nasal congestion, rhinorrhea, sore throat, and myalgia, with severe cases progressing to pneumonia, bronchiolitis, or respiratory failure, features that are indistinguishable from those seen in infections due to RSV, influenza, and other common respiratory pathogens [3,4,9,21,22,23]. Several studies have attempted to identify distinguishing features, but none have demonstrated pathognomonic findings. For example, wheezing and hypoxia are frequently observed in children with hMPV infection, but are also present in RSV and other viral infections [24,25]. In adults, hMPV may present with more influenza-like symptoms and a higher frequency of viral pneumonia compared to RSV, but these differences are not sufficient for clinical differentiation without laboratory confirmation [26]. Radiographic findings such as bronchial wall thickening have been described in hMPV pneumonia, yet these are not unique and can be seen with other viral etiologies [27]. Epidemiological patterns, such as a predilection for late winter to spring and higher incidence in young children and older adults, may aid in clinical suspicion but do not confer diagnostic specificity [4,9]. Ultimately, definitive diagnosis of hMPV infection requires laboratory testing, as clinical features alone cannot reliably distinguish it from other respiratory viruses [4,21,23,24,25,26].

Our findings show that children with hMPV infection had significantly more frequent nasal congestion (rhinorrhea), while adults experience more frequent dyspnea; this may reflect age-related differences in immune response, airway anatomy, and comorbidity burden. In children, the upper airway is relatively narrower and more reactive, and their immune response to hMPV tends to produce prominent upper respiratory symptoms such as rhinorrhea and nasal congestion. Pediatric airway epithelial cells also demonstrate increased mucus production and a stronger pro-inflammatory response to hMPV, which contributes to these symptoms [28]. In contrast, adults, especially the elderly and those with chronic lung or cardiac conditions, are predisposed to lower respiratory tract involvement. Dyspnea in adults is a more frequent occurrence due to decreased pulmonary reserve, age-related changes in lung function, and the higher likelihood of comorbidities such as COPD or heart failure, which exacerbate the impact of viral infection on gas exchange and respiratory mechanics [4,26,29]. hMPV infection in adults is associated with more severe lower respiratory tract symptoms, including dyspnea and pneumonia, particularly in those with underlying health conditions [4,26].

The changes in white blood cell (WBC) counts observed in our cohort were not specific to hMPV infection but were rather associated with disease severity. Elevated total leukocyte and neutrophil counts were more frequently observed in patients presenting with dyspnea or high-grade fever. The increased neutrophil counts among patients with dyspnea in our study are consistent with previous observations, as dyspnea often correlates with more severe disease or lower respiratory tract involvement, which is typically associated with a stronger neutrophilic inflammatory response [30,31,32,33]. Monocyte counts may also increase during hMPV infection, as antigen-presenting cells, including monocytes and macrophages, are activated, leading to their recruitment and activation as part of the innate immune response [34,35,36]. This monocytosis is not specific to hMPV infection and can also be observed in other viral respiratory infections; however, it represents a recognized feature of the inflammatory response associated with hMPV.

Overall outcomes were generally favorable in our study group, with no reported mortality. Adults more frequently developed respiratory failure requiring oxygen supplementation, and three adult cases required ICU admission, whereas children more often presented with moderate to severe dehydration and otitis. These findings indicate that the clinical outcomes of hMPV infection differ according to age group. These patterns are consistent with known age-related differences in immune response, airway anatomy, and comorbidity burden. Risk factors for severe disease in adults include advanced age, frailty, and underlying chronic cardiac or pulmonary conditions, while in children, prematurity and underlying conditions such as immunodeficiency or chronic lung disease are associated with an increased risk of severe outcomes [4,5,24,37,38,39].

The multivariable model explained a moderate proportion of the variance in length of hospital stay, which is consistent with the multifactorial nature of hospitalization duration in acute respiratory infections. Length of stay is influenced not only by clinical severity at presentation, but also by factors that cannot be captured in surveillance-based analyses, including institutional discharge practices, availability of outpatient support, and social or organizational considerations. Importantly, the objective of the model was to identify clinically meaningful independent associations rather than to achieve maximal predictive performance. In this context, the identification of dyspnea at admission as a strong and independent determinant of prolonged hospitalization supports its clinical relevance for early risk stratification.

Supportive care is the cornerstone of management for severe hMPV infection. This includes supplemental oxygen for hypoxemia, escalation to noninvasive or invasive mechanical ventilation for respiratory failure, and ICU-level monitoring and support as indicated. Management of pneumonia is also supportive and addresses disease pathogenesis, empiric antibiotics being considered only if bacterial superinfection is suspected. There are currently no approved antiviral therapies or vaccines for hMPV; investigational agents such as ribavirin and monoclonal antibodies have been used in select cases, but their efficacy remains unproven and they are not recommended for routine use [40,41,42,43,44]. In adults, careful management of comorbidities and monitoring cardiac complications is essential, as these are common contributors to morbidity and mortality in this population [5,45,46,47,48,49]. For patients presenting with dehydration, intravenous or oral rehydration is indicated. Otitis media is managed according to guidelines: antibiotics are reserved for cases with evidence of bacterial superinfection, while most cases of viral otitis resolve following anti-inflammatory treatment, without antibiotics [16,24,50,51].

In the second part of our study, we analyzed the genetic variability of hMPV based on the F protein. The genetic variability of the hMPV F gene was investigated due to the high immunogenicity of the F protein, the ability to elicit protective immune response, and the relevance as a potential target for vaccine development, monoclonal antibodies, and antiviral therapies [52]. Although the F protein is generally conserved, specific mutations may alter its structure and enhance the virus’s ability to infect host cells. For example, previous studies have shown that certain mutations in the F protein can increase fusion activity, leading to enhanced viral replication and, consequently, increased virulence [53]. Due to its evolutionary conservation, the F protein represents a suitable marker for phylogenetic classification into distinct clades [54]. Phylogenetic analysis of the Romanian hMPV sequences suggested a predominance of different subclades across the two consecutive seasons. hMPV strains detected in our region during the 2023–2024 season clustered within the A2b1 clade, together with reference sequences from Austria, China, and the United States. In contrast, strains identified during the 2024–2025 season clustered within the A2b2 clade, alongside reference sequences from the Netherlands, Japan, and the United States.

This genetic classification may provide insight into the evolutionary dynamics and transmission patterns of hMPV, with clade shifts indicating changes in circulating viral populations, possibly driven by immune selection, viral fitness, or epidemiological factors [4,55,56]. Genetic monitoring of hMPV clades may be valuable for tracking viral evolution, understanding epidemiological trends, and informing vaccine and therapeutic development. Surveillance enables detection of emerging variants with altered antigenicity or transmission potential, supports outbreak investigation, and provides data for public health responses. Although clinical outcomes are not strongly linked to clade, ongoing monitoring is essential as shifts in dominant clades may affect population immunity and future vaccine efficacy [4,55,56,57].

Our study has a set of limitations that should be considered when interpreting the results. First, the analysis was conducted at a single tertiary infectious diseases center, which may limit the external generalizability of the findings at national level or beyond. Clinical practices, admission criteria, and circulating viral strains may differ in other settings or other countries. Second, the study focused specifically on patients hospitalized with ILI or SARI, thereby leaving out mild or atypical hMPV infections; consequently, if not carefully interpreted, the findings may overestimate disease severity and complication rates. Third, the overall sample size was relatively limited, and children comprised a large part of the study group, which may inadvertently amplify observed age-related differences or mask heterogeneity within age categories. For the molecular analysis, only a subset of samples with high viral load could be successfully sequenced. Consequently, findings related to the distribution of hMPV clades should be interpreted as exploratory, providing initial insights into the circulation of viral genotypes in Romania, but not allowing definitive conclusions regarding molecular epidemiology or associations between viral clades and clinical severity.

The study also has several important strengths. NIID is the main national reference center for infectious diseases in Romania and a long-standing participant in the international Global Influenza Hospital Surveillance Network, ensuring application of standardized case definitions, as well as uniform diagnostic and data collection procedures. Case inclusion followed a rigorously standardized surveillance protocol that was applied continuously throughout two consecutive respiratory seasons, rather than being limited to peak epidemic periods. This continuous, prospective approach enhances the robustness, internal validity, and comparability of the data over time, reduces selection bias related to seasonal testing practices, and supports the reliability of the observed clinical and epidemiological patterns.

## 5. Conclusions

Our data provide a clinical and molecular characterization of hMPV infection among hospitalized patients in Romania. The results show that, although the clinical presentation of hMPV infection is nonspecific and largely overlaps with that of other viral respiratory infections, hospitalized adults more frequently developed respiratory failure requiring supplemental oxygen compared to children. Dyspnea at admission was identified as a factor strongly associated with prolongation of hospital stay, underscoring the importance of early clinical assessment and close monitoring of patients with signs of respiratory involvement, which may aid in risk stratification and optimization of clinical management from the early stages of hospitalization.

From a molecular perspective, the available data suggest the circulation of different hMPV subclades across consecutive seasons, supporting the dynamic evolutionary pattern of the virus and highlighting the need for ongoing genetic surveillance. Although the clinical implications of clade differences remain limited at present, molecular surveillance may become an important tool for improving the understanding of hMPV epidemiology and for informing future prevention and intervention strategies, including the development of vaccines or antiviral therapies. Overall, these findings support the inclusion of human metapneumovirus in the differential diagnostic strategies for severe acute respiratory infections and reinforce the role of integrated clinical and molecular surveillance in the management of viral respiratory infections in the post-pandemic era.

## Figures and Tables

**Table 1:** Clinical characteristics of hMPV infection and comparison according to age group.

Symptoms	All Patients	Children	Adults	Children vs. Adults	
	N = 71, n (%)	N = 44, n (%)	N = 27, n (%)	*p*-Value	OR (95%CI)
Fever	57 (80.3)	36 (81.8)	21 (77.7)	0.680	1.28 (0.39, 4.21)
Cough	62 (87.3)	36 (81.8)	26 (96.3)	0.139	0.17 (0.02, 1.47)
Nasal congestion	34 (47.9)	28 (63.6)	6 (22.2)	<0.001	6.12 (2.05, 18.32)
Dyspnea	21 (29.6)	6 (13.6)	15 (55.6)	0.001	0.12 (0.04, 0.39)
Sore throat	10 (14.1)	4 (9.1)	6 (22.2)	0.164	0.35 (0.09, 1.38)
Myalgia	8 (11.3)	0 (0.0)	8 (29.6)	NA	NA
Headache	12 (16.9)	0 (0.0)	12 (44.4)	NA	NA
Malaise	42 (59.2)	28 (63.6)	14 (51.9)	0.327	1.62 (0.61, 4.30)

NA, not applicable; OR, odds ratio; 95%CI, 95% confidence interval.

**Table 2:** Characteristics of laboratory parameters in hMPV infection and comparisons according to age group.

Lab Parameter	All Patients	Children	Adults	Children vs. Adults	
	N = 71	N = 44	N = 27	*p*-Value	OR (95%CI)/U (*r*)
WBC count (cells/μL), median (IQR)	8300 (5810, 12,230)	9010 (6720, 13,800)	6970 (4800, 8800)	0.013	281.5 (0.316)
Increased WBC, n (%)	15 (21.1)	11 (25.0)	4 (14.8)	0.293	1.92 (0.54, 6.77)
Decreased WBC, n (%)	6 (8.5)	4 (9.1)	2 (7.4)	0.963	1.25 (0.21, 7.33)
Neutrophils count (cells/μL), median (IQR)	4450 (3240, 6870)	4640 (3240, 7360)	4240 (3020, 6250)	0.603	414.5 (0.067)
Increased neutrophils, n (%)	15 (21.1)	11 (25.0)	4 (14.8)	0.293	1.92 (0.54, 6.77)
Decreased neutrophils, n (%)	4 (5.6)	3 (6.8)	1 (3.7)	0.782	1.90 (0.19, 19.28)
Monocytes count (cells/μL), median (IQR)	600 (420, 1200)	920 (580, 1380)	480 (340, 730)	<0.001	188 (0.492)
Increased monocytes	30 (42.3)	24 (54.5)	6 (22.2)	0.007	4.20 (1.42, 12.42)
Lymphocytes count (cells/μL), median (IQR)	1790 (1100, 3000)	2720 (1450, 4130)	1070 (590, 1740)	<0.001	142.5 (0.577)
Increased lymphocytes, n (%)	0 (0.0)	0 (0.0)	0 (0.0)	NA	NA
Decreased lymphocytes, n (%)	39 (54.9)	24 (54.5)	15 (55.5)	0.920	0.96 (0.36, 2.51)
Platelets count (×10^3^ cells/μL), median (IQR)	278 (201, 328)	309 (223, 387)	198 (152, 271)	<0.001	170.5 (0.525)
Increased platelets, n (%)	8 (11.3)	7 (15.9)	1 (3.7)	0.158	4.92 (0.57, 42.41)
Decreased platelets, n (%)	19 (26.8)	4 (9.1)	15 (55.6)	<0.001	0.08 (0.02–0.29)
Hemoglobin value (g/mL), median (IQR)	12.4 (11.7, 13.3)	12.2 (11.8, 12.7)	12.8 (11.1, 14.1)	0.284	377 (0.137)
Anemia	20 (28.2)	10 (22.7)	10 (37.0)	0.206	0.50 (0.17, 1.43)
CRP value (mg/L), median (IQR)	9.9 (3.6, 33.9)	6.9 (1.9, 21.1)	23.7 (7.2, 73.4)	<0.001	144 (0.387)
Increased CRP, n (%)	57 (80.3)	31 (70.5)	26 (96.3)	0.007	0.09 (0.01, 0.75)

CRP, C-reactive protein; IQR, interquartile range; NA, not applicable; OR, odds ratio; *r*, effect size; WBC, white blood cells; U, Mann-Whitney U test; 95%CI, 95% confidence interval.

**Table 3:** Outcome of hMPV infection among children and adults.

Symptoms	All Patients	Children	Adults	Children vs. Adults	
	N = 71, n (%)	N = 44, n (%)	N = 27, n (%)	*p*-Value	OR (95%CI)
Respiratory failure (requiring O_2_ supplementation)	17 (23.9)	3 (6.8)	14 (51.9)	<0.001	0.07 (0.02–0.27)
Moderate/severe dehydration (requiring IV fluids)	58 (81.7)	40 (90.1)	18 (66.7)	<0.001	5.00 (1.36–18.39)
Interstitial pneumonia ^#^	19 (26.8)	11 (25.0)	8 (29.6)	0.671	0.79 (0.27–2.31)
Pulmonary consolidation ^#^	3 (4.2)	0 (0.0)	3 (11.1)	NA	NA
Acute otitis media	4 (5.6)	4 (9.1)	0 (0.0)	NA	NA
ICU admission	3 (4.2)	0 (0.0)	3 (11.1)	NA	NA

^#^, X-ray diagnosis; ICU, intensive care unit; IV, intravenous; NA, not applicable; OR, odds ratio; 95%CI, 95% confidence interval.

## Data Availability

The dataset generated and analyzed during the current study are available from the corresponding author upon reasonable request.
